# lncRNA Expression Reveals the Potential Regulatory Roles in Hepatocyte Proliferation during Rat Liver Regeneration

**DOI:** 10.1155/2019/8597953

**Published:** 2019-11-11

**Authors:** Haijing Bai, Wei Jin, Jianlin Guo, Yi Ding, Cuifang Chang, Xueqiang Guo, Yaping Song, Jingbo Zhang, Cunshuan Xu

**Affiliations:** ^1^College of Life Science, Henan Normal University, Xinxiang 453007, Henan Province, China; ^2^State Key Laboratory Cultivation Base for Cell Differentiation Regulation and Henan Engineering Laboratory for Bioengineering and Drug Development, Xinxiang 453007, Henan Province, China; ^3^Institute of Basic Medical Sciences, Chinese Academy of Medical Sciences, Beijing 100005, China

## Abstract

Liver regeneration is a tissue growth process after loss or injury of liver tissue, which is a compensatory hyperplasia rather than true regeneration, mainly depending on hepatocyte proliferation. Currently, a large number of studies on hepatocyte proliferation have been conducted. However, studies on the regulation of long noncoding RNA (lncRNA) on hepatocyte proliferation are still limited. To identify specially expressed lncRNA during rat liver regeneration, high-throughput sequencing technology was performed, and a total of 2446 lncRNAs and 4091 mRNAs were identified as significantly differentially expressed. Gene ontology (GO) enrichment analysis was performed to analyze the role of differentially expressed mRNAs, and 695 mRNAs were identified to be related to cell proliferation. Then, an lncRNA-mRNA coexpression network based on the differentially expressed lncRNAs and proliferation-related genes was constructed to analyze the potential function of lncRNAs on hepatocyte proliferation, and ten lncRNAs, NONRATT003557.2, NONRATT005357.2, NONRATT003292.2, NONRATT001466.2, NONRATT003289.2, NONRATT001047.2, NONRATT005180.2, NONRATT004419.2, NONRATT005336.2, and NONRATT005335.2, were selected as key regulatory factors, which may play crucial roles in hepatocyte proliferation during rat liver regeneration. Finally, a protein-protein interaction (PPI) network was established to illuminate the interaction between proliferation-related genes, and ten hub genes (Aurkb, Cdk1, Cdc20, Bub1b, Mad2l1, Kif11, Prc1, Ccna2, Top2a, and Ccnb1) were screened with the MCC method in the PPI network, which may be important biomarkers involved in the hepatocyte proliferation during rat liver regeneration. These results may provide clues for a more comprehensive understanding of the molecular mechanism of hepatocyte proliferation during rat liver regeneration.

## 1. Introduction

The vast majority of the eukaryotic genomes are transcribed into noncoding RNAs, which can be divided into small noncoding RNAs (<200 bp) and long noncoding RNAs (lncRNAs; ≥200 nt) based on transcript size [1]. lncRNAs can be divided into five categories: sense, antisense, bidirectional, intronic, and intergenic [2]. Initially, lncRNAs were considered to be “dark matter,” as byproduct of transcription of RNA polymerase II, with no biological function. In the past 20 years, genome-wide identification of lncRNAs has become possible with the development of high-throughput technology of RNA-seq, many of which are involved in various biological functions [3]. Increasing lncRNAs have been found to play a critical role in biological processes, like development [4], gene transcriptional regulation [5], chromatin regulation [6], epithelial-to-mesenchymal transition (EMT) [7], and cell proliferation [8].

In rodents and humans, the liver can grow rapidly after partial hepatectomy (PH) or acute chemical injury. This growth process is known as LR, which is a compensatory hyperplasia rather than true regeneration [9]. During LR, quiescent hepatocytes undergo one or two rounds of replication and then return to a nonproliferative state [10]. This process is very complex and regulated by a variety of growth factors, cytokines and noncoding RNAs [11, 12]. Therefore, the study of the molecular mechanism of hepatocyte proliferation is crucially important to understand the process of LR and provide clues for the treatment of liver diseases. Several recent studies have shown that lncRNAs play a critical role in hepatocyte proliferation [12–14]. However, the study of hepatocyte proliferation during LR is still largely unknown.

In the present study, high-throughput sequencing technology was used to identify DE lncRNAs and mRNAs during rat LR. Then, functional enrichment analysis of DE mRNAs was performed to screen proliferation-related genes. Finally, the lncRNA-mRNA coexpression network and PPI network were constructed based on DE lncRNAs and proliferation-related genes to elucidate the molecular mechanism of hepatocyte proliferation during LR. These results lay a foundation for understanding the regulatory function of lncRNAs on hepatocyte proliferation and provide an important clue for the study of the LR process.

## 2. Materials and Methods

### 2.1. Preparation of 2/3 Hepatectomy Model

The healthy adult male Sprague Dawley (SD) rats weighing 210∼250 g were provided by the Laboratory Animal Center of Zhengzhou University (Zhengzhou, China). These rats were raised in a controlled temperature room of 19∼23°C with a relative humidity of 50∼70% and an illumination time of 12 h/d (8 : 00 to 20 : 00) and permitted to freely have water and food. A total of 60 rats were taken for the experiment with six rats per group: nine PH groups and one normal group (CG). The rats in PH groups were conducted 2/3 PH according to the method of Xu et al. The rats were anesthetized and condemned to death at 0, 2, 6, 12, 24, 30, 36, 72, 120, and 168 h after operation. The right liver lobes of six rats were mixed at each time point and stored at −80°C. All operations conformed to the Animal Protection Law of China and Animal Ethics.

### 2.2. RNA Sequencing

RNA sequencing was performed by the Shanghai OE Biotech (Shanghai, China). In brief, the mirVana miRNA Isolation Kit (Ambion) was used to extract the total RNA from liver tissues. The TruSeq Stranded Total RNA with Ribo-Zero Gold (Illumina) was used to construct cDNA libraries. The purified cDNA libraries were sequenced on Illumina HiSeq 2500 following the manufacturer's instruction. After filtrating the adaptor and low-quality reads, clean reads were obtained for subsequent analysis. The reads were matched to the rat reference genome using the hisat2 (v2.2.1.0) software. The StringTie2 (v1.3.3b) software was used to splice the aligned reads. lncRNA identification included two categories: one is known lncRNA, which completely matches with the known lncRNAs, and the other is candidate lncRNA lacking protein-coding ability, whose length is greater than 200 bp and exon is greater than or equal to 2. The software CPC (v0.9-r2), CNCI (v1.0), Pfam (v30), and PLEK (v1.2) were used to predict the protein-coding ability of transcripts. The expression of transcription was calculated by the fragments per kilobase of exon per million reads mapped (FPKM) method using the bowtie2 (v2.2.9) and eXpress (v1.5.1) software.

### 2.3. Identification of Differentially Expressed lncRNAs

The counts of lncRNAs in each sample were standardized by the baseMean value using the DESeq (1.18.0) software. Differentially expressed (DE) lncRNAs were identified with fold change ≥2 or ≤0.5 and *p* < 0.05 as the threshold. All DE lncRNAs in nine PH groups underwent hierarchical clustering analysis using the cluster3.0 and treeview software.

### 2.4. GO Enrichment Analysis

Gene ontology (GO) enrichment analysis of the DE mRNAs was conducted using David Bioinformatics Resources 6.8 (https://david.ncifcrf.gov/). The enrichment analysis consisted of three parts: biological process (BP), molecular function (MF), and cellular component (CC). *p* < 0.05 is considered statistically significant, which was calculated by the EASE score.

### 2.5. lncRNA-mRNA Coexpression Analysis

To explore the relationship between DE lncRNAs and proliferation-related genes, a coexpression analysis was performed. Pearson's correlation coefficients (PCCs) were calculated between the DE lncRNAs and the proliferation-related genes, and only lncRNA-mRNA pairs with PCC ≥0.8 and *p* ≤ 0.05 were selected and considered as coexpression. Then, these lncRNA-mRNA pairs were used to construct a coexpression network, which was visualized by the Cytoscape v3.6.1 software. The node degree was determined by the number of directly connected neighbors to the topological property of the network.

### 2.6. Construction of PPI Network and Screening of the Key Gene

To illustrate interactions between proliferation-related DE mRNAs, the string database (https://string-db.org/) was used to construct a protein-protein interaction (PPI) network. Only the interacting pairs with combined score ≥0.4 were selected and considered to be significant. The PPI network was visualized by using the Cytoscape v3.6.1 software. Then, a Cytoscape plugin cytoHubba was used to identify the key genes adopting the MCC method.

### 2.7. RT-PCR Validation

RNA-seq results were validated by RT-PCR, and the primers are listed in Table 1. cDNA was synthesized using the cDNA Reverse Transcription Kit (Takara, Tokyo, Japan). The qRT-PCR was performed using Q-SYBR Green Supermix (Bio-Rad). Primers were also designed to amplify *β*-actin as an endogenous control. The expression of each lncRNA was represented as fold change using 2^−ΔΔCt^ methods.

## 3. Results

### 3.1. Sequencing and Identification of lncRNAs during Rat LR

To identify the expression of lncRNAs during rat LR, 10 cDNA libraries were constructed from the regeneration rat liver at different time points after surgery. The Illumina HiSeq X Ten platform was used to sequence these cDNA libraries, and a total of 1116M raw reads were produced. After filtering adaptor sequences and low-quality reads, 1084.09M clean reads were obtained. The percentage of clean reads varied from 90.73 to 95.29%, and the percentage of GC content varied from 48.77 to 51.08% in each library. Approximately 95.91–97.83% of clean reads were selected for further research after mapping the clean reads to the rat reference genome (Table 2).

### 3.2. Identification of DE lncRNAs and DE mRNAs

**Figure 1:**
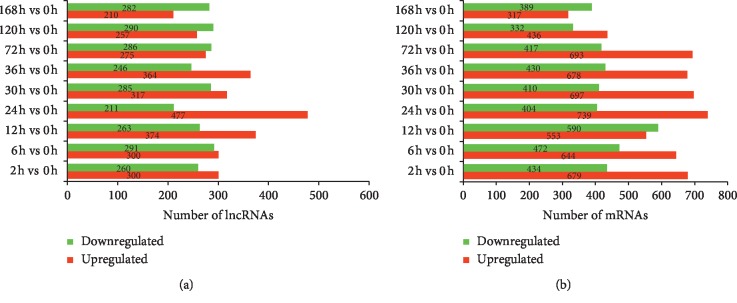
Number of DE lncRNAs and DE mRNAs at different time points during rat LR. (a) Number of upregulated and downregulated lncRNAs at nine time points. (b) Number of upregulated and downregulated mRNAs at nine time points.

**Figure 2:**
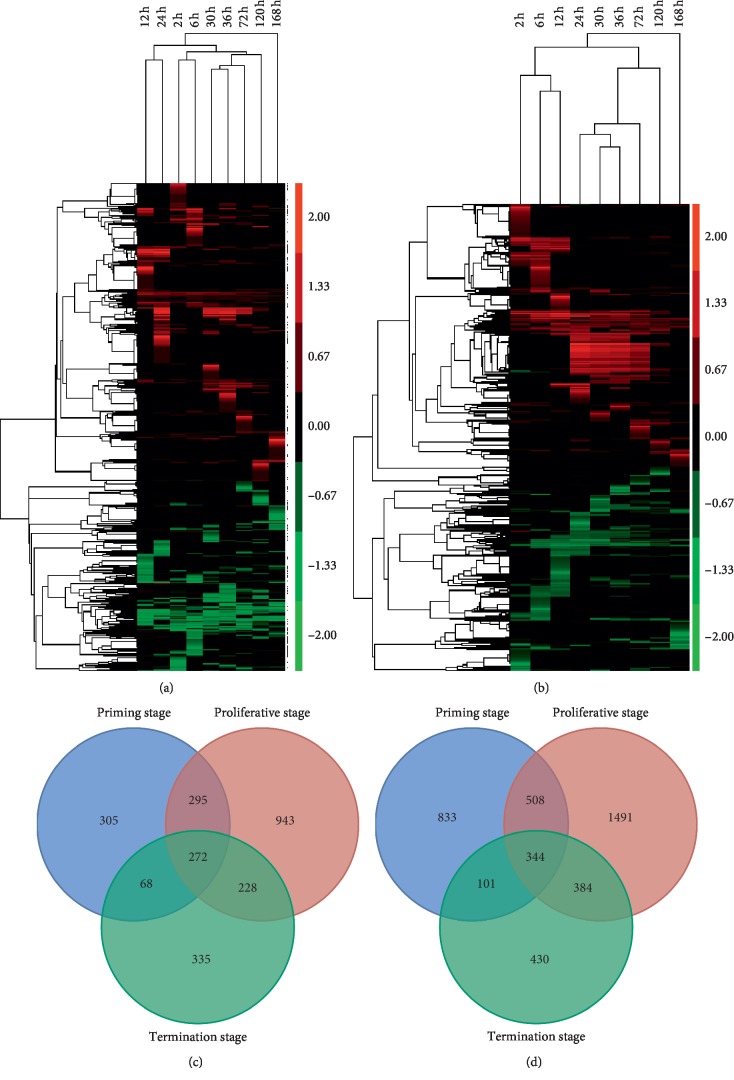
Analyses of DE lncRNAs in the RNA-seq libraries. (a) Hierarchical clustering analysis of DE lncRNAs at nine time points of rat LR. (b) Hierarchical clustering analysis of DE mRNAs at nine time points of rat LR. (c) Venn diagram showing the DE lncRNAs at three stages of rat LR. (d) Venn diagram showing the DE mRNAs at three stages of rat LR.

The expression abundance of the lncRNAs was evaluated by FPKM (fragments per kB per million reads) using DESeq. A total of 2446 lncRNAs were determined to be differentially expressed during rat LR, with 1120 upregulated, 731 downregulated, and 595 up/downregulated lncRNAs (Figure 1(a) and [Supplementary-material supplementary-material-1]). To explore the similarity of gene expression, hierarchical clustering was adopted to analyze the expression of DE lncRNAs (Figure 2(a)). To further explore the interactions of DE lncRNAs at different stages: initial stage (2–6 h), proliferation stage (12–72 h), and termination stage (120–168 h), a Venn diagram was constructed using these DE lncRNAs (Figure 2(c)). Among them, 272 DE lncRNAs were common to all three stages.

Through high-throughput RNA-seq, the expression profile of 28635 mRNAs was measured. Among them, 4091 mRNAs were found to be differentially expressed, of which 2,256 were upregulated, 1,686 were downregulated, and 149 were up/downregulated (Figure 1(b) and [Supplementary-material supplementary-material-1]). Hierarchical clustering was employed to analyze the expression similarity of DE mRNAs (Figure 2(b)). Venn analysis was conducted to explore the differences of DE mRNAs at different stages (Figure 2(d)).

### 3.3. GO and KEGG Enrichment Analysis

**Figure 3:**
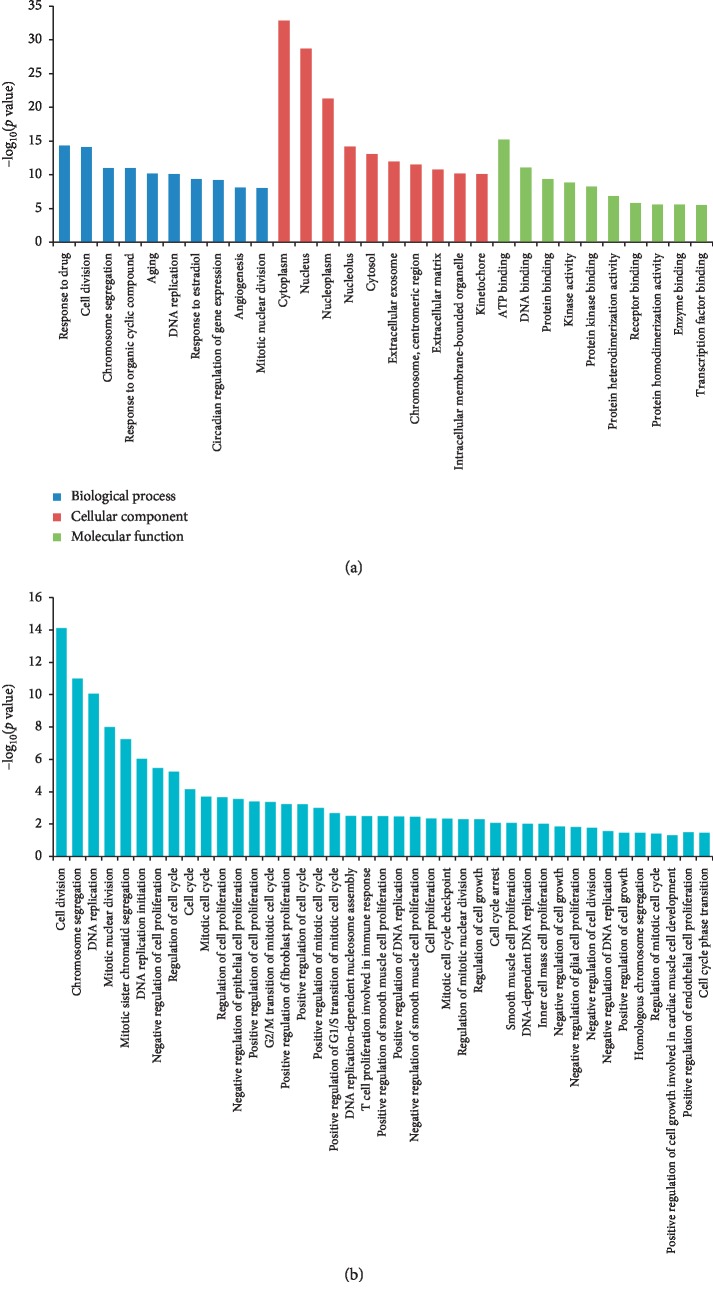
GO enrichment analysis of the DE mRNAs. (a) The top 30 significant GO terms in GO enrichment analysis at *p* value <0.05. Green represents biological processes, red represents cellular components, and blue represents molecular functions. (b) GO terms associated with cell proliferation at the biological process level.

GO enrichment analysis was employed to determine the function of deregulated genes during rat LR, and 689 significant GO terms (451 under BP, 116 under CC, and 122 under MF) were enriched. The top 30 GO terms of the three groups are listed in Figure 3(a) ([Supplementary-material supplementary-material-1]). The most enriched BP terms were response to drug, cell division, and chromosome segregation. As for CC, the most enriched terms were cytoplasm, nucleus, and nucleoplasm. The most enriched MF terms were related to binding activity. Of these BP terms, 41 were associated with cell proliferation involving 695 mRNAs, corresponding to 585 genes (Figure 3(b) and [Supplementary-material supplementary-material-1]).

### 3.4. Coexpression Network Construction Based on DE lncRNAs and Proliferation-Related DE mRNAs

**Figure 4:**
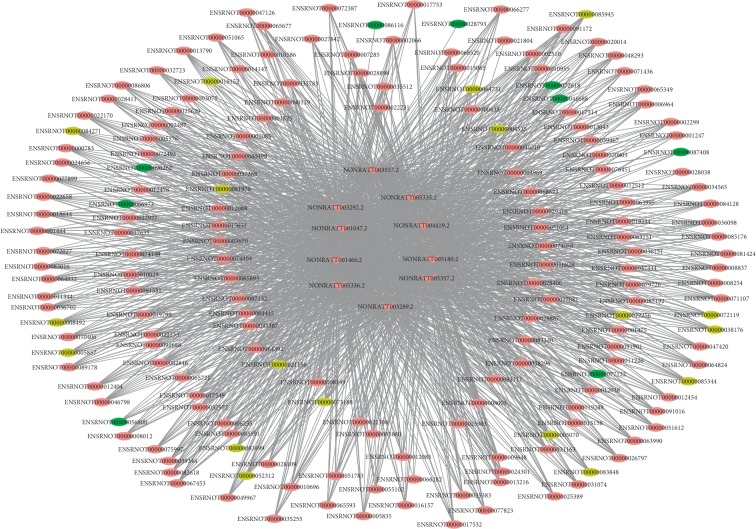
The top 10 lncRNAs and their interaction mRNAs. V and ellipse represent lncRNAs and mRNAs, respectively. Upregulated genes are labeled in red, downregulated genes are labeled in green, and up/downregulated genes are labeled in yellow.

The coexpression analysis of the screened proliferation-related DE mRNAs and DE lncRNAs found 18343 significant coexpression pairs, of which 17547 pairs were positively correlated (PCC ≥ 0.8) and 796 pairs were negatively correlated (PCC ≤ −0.8) ([Supplementary-material supplementary-material-1]). Subsequently, a coexpressed network was constructed using screened lncRNA-mRNA pairs, and it was found that some lncRNAs could interact with multiple mRNAs. According to nodes and connectivity, the top 10 lncRNAs were selected, which may exert important regulatory roles in hepatocyte proliferation, lncRNAs NONRATT003557.2 (degree = 178), NONRATT005357.2 (degree = 178), NONRATT003292.2 (degree = 177), NONRATT001466.2 (degree = 176), NONRATT003289.2 (degree = 176), NONRATT001047.2 (degree = 175), NONRATT005180.2 (degree = 175), NONRATT004419.2 (degree = 173), NONRATT005336.2 (degree = 173), and NONRATT005335.2 (degree = 169) (Figure 4). The expression levels of the top 10 lncRNAs during rat LR are shown in Table 3. Among them, NONRATT005357.2 had the highest difference at 24 h in regenerating the liver, and the fold change was 119.

### 3.5. Construction of PPI Network Based on Proliferation-Related Genes

**Figure 5:**
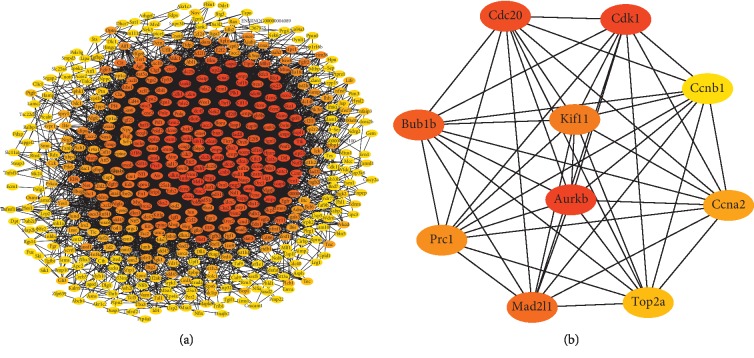
PPI network of proliferation-related genes. (a) PPI network of 551 genes. (b) Top 10 hub DE mRNAs of the PPI network. The node color changes gradually from yellow to red in the ascending order according to scores with the MCC method.

To better elucidate the interaction network of proliferation-related genes, a PPI network containing 551 nodes with scores greater than or equal to 0.4 was constructed using the string database (Figure 5(a)). Moreover, a cytoHubba plugin was used to select the top 10 key genes from the PPI network using the MCC method. The 10 key genes were Aurkb, Cdk1, Cdc20, Bub1b, Mad2l1, Kif11, Prc1, Ccna2, Top2a, and Ccnb1 (Figure 5(b)). The expression levels of the 10 key genes during rat LR are shown in Table 4. The median of multiple mRNAs corresponding to one gene is taken as its expression level. Among them, Cdk1 had the highest difference at 24 h in regenerating the liver, and the fold change was 109.

### 3.6. Quantitative Real-Time PCR Validation

**Figure 6:**
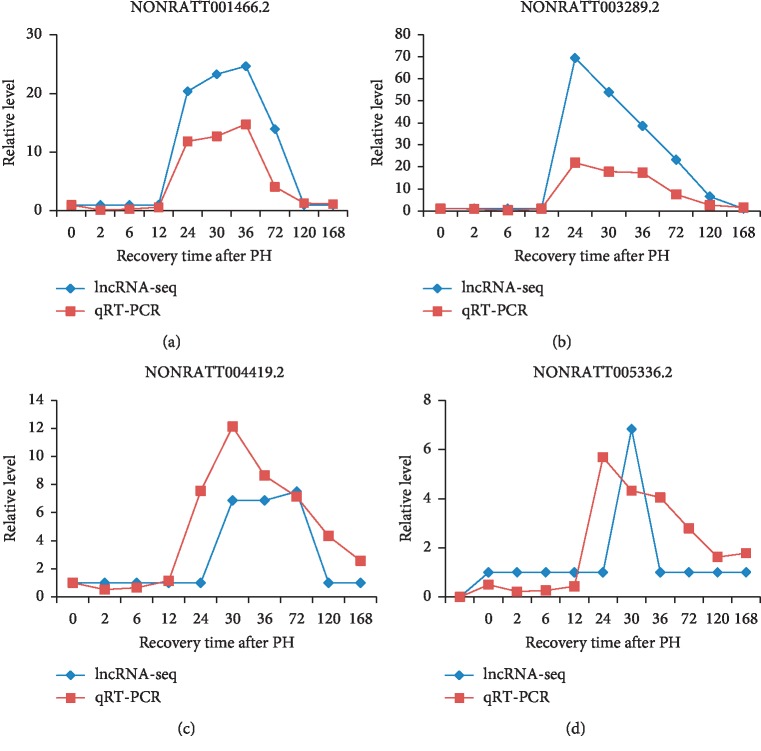
Validation of differentially expressed lncRNAs by qRT-PCR.

We validated the high-throughput RNA-seq results by performing qRT-PCR analysis of differentially expressed lncRNAs; the expression patterns revealed similar conclusions (Figure 6). Our RNA-seq results showed lncRNAs NONRATT003289.2, NONRATT001466.2, NONRATT004419.2, and NONRATT005336.2 were upregulated during rat LR.

## 4. Discussion

In rodents and humans, the liver can grow rapidly after partial hepatectomy or acute chemical injury. This growth process is known as LR, which is a compensatory hyperplasia rather than true regeneration, mainly depending on the proliferation of hepatocytes [9]. To explore the regulatory roles of lncRNAs in hepatocyte proliferation during rat LR, high-throughput RNA-seq was performed to identify lncRNAs and mRNAs. In the present research, 2446 DE lncRNAs and 4091 DE mRNAs were identified. To investigate the function of DE mRNAs during rat LR, GO enrichment analysis was performed. The result indicated that a large number of GO terms were associated with response to stress, cell proliferation, oxidation-reduction, regulation of transcription, metabolism, and apoptosis, which were considered to be important activities in LR [15–17]. Of these GO terms, 41 were associated with cell proliferation, involving 695 mRNAs.

Through coexpression network analysis, a total of 18343 coexpression pairs were obtained based on DE lncRNAs and proliferation-related DE mRNAs. Among these coexpression pairs, 95.7% were positively correlated and 4.3% were negatively correlated. These results indicate that lncRNAs may regulate the expression of genes mainly in a positive way. It was also found that some lncRNAs could be coexpressed with multiple mRNAs, suggesting that lncRNAs could regulate multiple mRNAs. According to nodes and connections, the top 10 lncRNAs (NONRATT003557.2, NONRATT005357.2, NONRATT003292.2, NONRATT001466.2, NONRATT003289.2, NONRATT001047.2, NONRATT005180.2, NONRATT004419.2, NONRATT005336.2, and NONRATT005335.2) were selected. These lncRNAs were connected to 158 common DE mRNAs, respectively, such as Slc25a16, Cdk1, Rfc3, Brca2, Mcm7, Cdca7, Hat1, Chaf1b, Rfc4, and Mcm4. These findings indicated that these lncRNAs and their interaction genes may play an important role in hepatocyte proliferation during rat LR.

cytoHubba, a Cytoscape plugin, could provide 11 topological analysis methods to explore important nodes in biological networks including maximal clique centrality (MCC). MCC, a new method, has a better precision in identifying hub proteins. Through the MCC method, 10 hub mRNAs were selected including Aurkb, Cdk1, Cdc20, Bub1b, Mad2l1, Kif11, Prc1, Ccna2, Top2a, and Ccnb1. Aurkb (aurora kinase B) was involved in regulation of chromatin, glycolysis metabolism, regulation of telomerase activity, regulation of zygote development, and turnover of kinetochore microtubules [18–22]. It was also associated with AKT signaling pathway, MAPK signaling pathway, inflammatory pathway, DNA damage response pathway, Wnt signaling pathway, and PI3K/Akt/NF-*κ*B signaling pathway [21, 23–27]. Cdk1 (cyclin-dependent kinase 1) was involved in cell cycle, mitosis, DNA end resection, homologous recombination, DNA damage checkpoint, anaphase spindle dynamics, cytokinesis, chromosome biorientation, apoptosis, cell proliferation and survival, and DNA synthesis [28–35]. It also played an important role in AMPK signaling pathway, AKT signaling pathway, Golgi checkpoint signaling, PDK1-PI3K/Akt signaling pathway, type I interferon signaling, and p53 signaling pathway [36–40]. Cdc20 (cell division cycle 20), an APC activator protein, could regulate mitosis, cell cycle, and presynaptic differentiation [41–44]. It was also involved in Wnt/*β*-catenin signaling pathway and p38 MAPK signaling pathway. Bub1b (BUB1 mitotic checkpoint serine/threonine kinase B) and Mad2l1 (mitotic arrest deficient 2 like 1) were involved in the spindle checkpoint during mitosis [45–48]. Kif11 (kinesin family member 11), a microtubule motor, played a vital role in regulating the transport of *β*-actin mRNA and cell motility through physically interacting with ZBP1, which could govern the direction of migration by responding to directional cues in chemotaxis [49, 50]. Prc1 (protein regulator of cytokinesis 1), a cell cycle protein, played an important role in ensuring proper cell division through directly acting on protein (FIP), and P53 could regulate the transcription of Prc1 [51, 52]. Ccna2 (cyclin A2) was a key factor in cell cycle, and Ccna2 repression regulated by miR-22 could inhibit HCC cell proliferation and tumorigenesis [53, 54]. It was also a prognostic biomarker for several cancers, such as ER+ breast cancer, pancreatic ductal adenocarcinoma, and colorectal cancer [55–57]. Top2a (DNA topoisomerase II alpha), a key enzyme in DNA replication, was recruited to ultrafine anaphase bridges (UFBs) by TopBP1 to ensure faithful separation of sister chromatids, and these proteins played an important role in maintaining genome stability [58]. Top2a was overexpressed in hepatocellular carcinoma and associated with early age onset, shorter patient survival, and chemoresistance [59]. Ccnb1 (cyclin B1) was a key factor in cell cycle and regulated by STAT3 via the E2F modulating G2-M phase checkpoint [60]. High expression of Ccnb1 was associated with poor prognosis in HCC patients, and knockdown of Ccnb1 could significantly inhibit cell proliferation, migration, and invasion in HCC [61]. Therefore, these genes may play a vital role in hepatocyte proliferation during LR.

## 5. Conclusions

In this study, the comprehensive expression abundance of lncRNAs and mRNAs was identified by RNA-seq analysis during rat LR. The lncRNA-mRNA coexpression network and PPI network based on lncRNAs and proliferation-related genes were constructed, and 10 key lncRNAs and 10 key mRNAs were determined that may play crucial roles in hepatocyte proliferation. Our study provides a new idea to better understand the mechanism of LR.

## Figures and Tables

**Table 1:** Primer sequence.

Gene name	Sense primer	Antisense primer	Product length
NONRATT003289.2	AATGCCAGGCCATGCTAAGGAC	GCTCTGCCAGGTGACTGCTTC	180
NONRATT001466.2	TCTGCTGTTGACATTGGCGAAGG	CTAGCATGTGAGAGGTGACGTGAC	189
NONRATT004419.2	AGCCTCCTGAGTCCTGGAATTCTG	GTGAGTCGTGAGTGAGCTGAAGTG	133
NONRATT005336.2	AAGCTCAACACTGCCTGAGTCTTC	GCATGAGCCTTGGAGGACATCTG	112

**Table 2:** Overview of RNA-seq.

Sample	raw_reads	raw_bases	clean_reads	clean_bases	valid_bases (%)	GC (%)	Mapped reads
0 h	119.65M	17.95G	115.99M	17.05G	94.99	50.92	111716504 (96.32%)
2 h	120.14M	18.02G	116.69M	17.17G	95.25	50.57	113657852 (97.40%)
6 h	119.57M	17.94G	116.13M	17.09G	95.29	50.26	113238482 (97.51%)
12 h	119.91M	17.99G	117.68M	16.68G	92.75	49.88	114912884 (97.65%)
24 h	119.27M	17.89G	117.28M	16.23G	90.73	48.77	114629815 (97.74%)
30 h	88.95M	13.34G	85.96M	12.64G	94.74	50.29	83564746 (97.21%)
36 h	120.15M	18.02G	116.44M	17.16G	95.22	50.62	113908105 (97.83%)
72 h	91.48M	13.72G	88.07M	12.9G	93.98	51.08	86032773 (97.68%)
120 h	120.82M	18.12G	116.34M	17.12G	94.47	50.97	112635056 (96.82%)
168 h	96.06M	14.41G	93.51M	13.73G	95.28	50.71	89681397 (95.91%)

**Table 3:** Expression levels of the top 10 lncRNAs during rat LR.

lncRNAs	0 h	2 h	6 h	12 h	24 h	30 h	36 h	72 h	120 h	168 h
NONRATT003557.2	1	1	1	1	**14.67**	**15.41**	**10.44**	1	1	1
NONRATT005357.2	1	1	1	**9.79**	**119.12**	**59.69**	**71.42**	**32.46**	1	1
NONRATT003292.2	1	1	1	1	**43.68**	**40.42**	**28.50**	**18.78**	1	1
NONRATT001466.2	1	1	1	1	**20.37**	**23.28**	**24.66**	**13.95**	1	1
NONRATT003289.2	1	1	1	1	**69.44**	**53.97**	**38.69**	**23.26**	**6.55**	1
NONRATT001047.2	1	1	1	1	1	1	**4.86**	1	1	1
NONRATT005180.2	1	1	1	1	**9.81**	**9.02**	**10.33**	1	1	1
NONRATT004419.2	1	1	1	1	1	**6.87**	**6.87**	**7.5**	1	1
NONRATT005336.2	1	1	1	1	1	**6.83**	1	1	1	1
NONRATT005335.2	1	1	1	1	**6.89**	**6.44**	**5.27**	1	1	1

Text in bold denotes the expression level higher than the control.

**Table 4:** Expression levels of 10 key genes during rat LR.

Genes	0 h	2 h	6 h	12 h	24 h	30 h	36 h	72 h	120 h	168 h
Aurkb	1	*0.02*	1	1	**41.88**	**26.57**	**23.96**	**14.00**	**3.72**	1
Cdk1	1	1	1	1	**109.01**	**66.43**	**80.87**	**42.31**	**3.63**	1
Cdc20	1	1	1	1	**36.85**	**27.36**	**25.84**	**13.01**	**2.83**	1
Bub1b	1	1	1	1	**38.34**	**26.78**	**22.37**	**12.92**	**5.08**	1
Mad2l1	1	1	1	1	**20.26**	**13.71**	**13.11**	**7.02**	1	1
Kif11	1	1	1	1	**20.48**	**13.24**	**11.22**	**7.31**	**2.12**	1
Prc1	1	1	1	1	**46.88**	**29.75**	**27.15**	**16.27**	**3.59**	1
Ccna2	1	*0.05*	1	1	**49.97**	**31.85**	**30.16**	**15.45**	**3.92**	1
Ccnb1	1	1	1	1	**60.31**	**37.31**	**37.82**	**18.90**	**3.45**	1
Top2a	1	*0.18*	1	1	**49.62**	**34.69**	**23.24**	**14.75**	**3.03**	1

Text in bold and text in italics denote the expression level higher and lower than the control, respectively.

## Data Availability

The data used to support the findings of this study are included within the supplementary information file(s).

## Supplementary Materials

Supplementary Materials Table S1: results of analyzing differentially expressed lncRNAs at 2, 6, 12, 24, 30, 36, 72,120, and 168 h after PH was compared with CG. Table S2: results of analyzing differentially expressed mRNAs at 2, 6, 12, 24, 30, 36, 72,120, and 168 h after PH was compared with CG. Table S3: results of GO enrichment analysis of differentially expressed mRNAs. Table S4: screened coexpression pairs between DE lncRNAs and proliferation-related DE mRNAs based on the Pearson correlation coefficient.
